# Identification and validation of cellular senescence-related genes and immune cell infiltration characteristics in intervertebral disc degeneration

**DOI:** 10.3389/fimmu.2025.1589849

**Published:** 2025-05-29

**Authors:** Hao Li, Lei Miao, Jiayuan Wu, Ye Wang, Jiyuan Xia, Da He

**Affiliations:** ^1^ Department of Orthopedics, Beijing Jishuitan Hospital, Capital Medical University, Beijing, China; ^2^ School of Computer Science, South China Business College of Guangdong University of Foreign Studies, Guangzhou, China

**Keywords:** GEO database, intervertebral disc degeneration, cellular senescence, immune infiltration, MAPK1

## Abstract

**Objective:**

To identify cell senescence-related genes and immune cell infiltration characteristics in intervertebral disc degeneration (IVDD) using bioinformatics and investigate their biological functions and signaling pathways.

**Methods:**

GSE56081 and GSE23130 datasets were downloaded from the Gene Expression Omnibus (GEO) database. The former was used for the analysis of differentially expressed genes (DEGs) and the latter was used as the validation set. Intersection analysis of DEGs and cell senescence-related genes was performed to screen for senescence-related differentially expressed genes (SRDEGs). SRDEGs were analyzed using Gene Ontology (GO) and Kyoto Encyclopedia of Genes and Genomes (KEGG). A protein–protein interaction (PPI) network was also drawn, and the hub SRDEGs were obtained using 11 different algorithms. Immune infiltration analysis was then performed. Receiver operating characteristic (ROC) curve and quantitative polymerase chain reaction (qPCR) were used to evaluate the diagnostic value of the hub SRDEGs.

**Results:**

Four hub SRDEGs, SP1, FOXO1, ESR1, and MAPK1, were identified. SP1 was downregulated in IVDD, while the other three hub genes were upregulated. ROC curve verification showed that the AUC for SP1, FOXO1, ESR1, and MAPK1 were 0.483, 0.683, 0.683, and 0.725, respectively. RT-qPCR confirmed that MAPK1 expression was higher in the degeneration group (t = 3.229, P <0.001). Immune infiltration analysis demonstrated elevated proportions of CD8 T cells and activated NK cells in IVDD samples, with MAPK1 showing positive correlations with CD8 T cells, activated NK cells, and neutrophils but negative correlations with naive CD4 T cells, B memory cells, and resting NK cells.

**Conclusion:**

These findings highlight MAPK1 as a pivotal regulator of cellular senescence and immune cell infiltration in IVDD pathogenesis, offering novel therapeutic targets for intervention.

## 1 Introduction

Intervertebral disc degeneration (IVDD), which is highly age-related and can give rise to a host of spinal surgical diseases, such as lower back pain, disc herniation, spinal stenosis, and degenerative scoliosis, is the pathological basis of many degenerative spinal diseases. These conditions affect patients’ quality of life and impose a heavy burden on social healthcare (1–4). Moreover, with an increasing aging population, this will inevitably become a problem that needs to be urgently solved in today’s society. IVDD mainly includes the death of nucleus pulposus cells and degradation of the extracellular matrix, which is closely related to the senescence of nucleus pulposus cells (5–7).

Cellular senescence, which is characterized by irreversible cessation of the cell cycle, is a process in which cells gradually lose their ability to proliferate and differentiate and experience a decline in various physiological functions during the course of life (8). Although the cell cycle is arrested and proliferation ceases, senescent cells still possess metabolic activity and an enhanced secretory function. They influence the microenvironment of adjacent cells and tissues through autocrine and paracrine pathways, leading to and accelerating the occurrence of senescence-related diseases such as IVDD (4, 9, 10). Inflammatory responses, oxidative stress, and mitochondrial dysfunction can accelerate cellular senescence, thereby promoting intervertebral disc degeneration. However, the specific gene targets and molecular mechanisms related to cellular senescence in IVDD remain unclear. Immune cell infiltration is the accumulation and aggregation of immune cells in tissues, such as B cells, T cells, and macrophages. A previous study showed that when the physical barrier between the intervertebral disc and systemic circulation is damaged, various immune cells are involved in the inflammatory response or IVDD progression (11). The present study, through bioinformatics analysis methods, comprehensively explores the key roles of cellular senescence-related genes and immune cell infiltration in the onset and progression of IVDD, aiming to provide new diagnostic and therapeutic targets for IVDD.

## 2 Materials and methods

### 2.1 Data collection

The datasets GSE56081 and GSE23130 used in this study were obtained from the Gene Expression Omnibus (GEO) database (https://www.ncbi.nlm.nih.gov/geo/). Dataset GSE56081 (12), containing five degenerated nucleus pulposus samples and five non-degenerated ones, was used to identify differentially expressed genes (DEGs). Dataset GSE23130 (13), with eight degenerated and 15 non-degenerated nucleus pulposus samples, was the validation analysis dataset.

Cellular senescence-related genes were obtained from the CellAge online database (https://www.genomics.senescence.info/cells), total 866 in number (14).

### 2.2 Determination of DEGs and senescence-related DEGs

The DEGs in IVDD were acquired using the R package “limma,” and the criteria for identifying the DEGs were set at |log2 FC| >1 and adjusted p-value <0.05. A Venn diagram was used to intersect the DEGs from GSE56081 with the cellular senescence-related genes from the CellAge database to obtain senescence-related DEGs (SRDEGs). Volcano plots of DEGs and hierarchical clustering heatmaps of SRDEGs were generated using the R package “ggplot2.”

### 2.3 Functional enrichment analysis

The “clusterProfiler” package in R was used for GO and KEGG enrichment analyses of SRDEGs. GO enrichment analysis classified gene functions into three categories: Biological Process (BP), Cellular Component (CC), and Molecular Function (MF). KEGG enrichment analysis covers a wide range of biochemical processes, divided into seven main categories: metabolism, genetic information processing, environmental information processing, cellular processes, organismal systems, human diseases, and drug development. These processes are represented as pathway maps of molecular interactions and reactions. The threshold for significance was set at p <0.05. Enrichment analysis plots were visualized using the R package “ggplot2.”

### 2.4 Protein–protein interaction network analysis

PPI network analysis of SRDEGs was conducted using STRING (https://cn.string-db.org/), a common tool for assessing protein interactions. The results were then imported into Cytoscape v3.10.3 (https://cytoscape.org/) to construct a PPI network. In this network, the nodes represent SRDEGs and lines (connections between nodes) represent the interactions between different SRDEGs.

### 2.5 Screening and correlation analysis of the hub SRDEGs

Based on the constructed PPI network, 11 algorithms (CycloHubba MCC, DMNC, MNC, Degree, EPC, BottleNeck, EcCentricity, Closeness, Radiality, Betweenness, Stress) were used to calculate the top 30 SRDEGs. Different algorithms represent different topological features based on network analysis, and the selection of multiple algorithms can provide a more robust and comprehensive evaluation of potential hub genes from multiple network perspectives (15). The intersection of these results was considered the hub SRDEGs. Correlations between hub SRDEGs were assessed using Pearson correlation analysis. In Pearson correlation analysis, the r value refers to the correlation coefficient and is used to assess the effect size. A scatter plot of the correlation was drawn using the R package “ggplot2.”

### 2.6 Functional enrichment analysis of the hub SRDEGs

GO and KEGG enrichment analyses of the hub SRDEGs were performed using the R software package “clusterProfiler,” and the threshold p-value was set at <0.05. The R package “ggplot 2” was used for visualization of enrichment analysis plots.

### 2.7 Immune infiltration analysis by CIBERSORT algorithm

Immune cell infiltration in IVDD and control samples was evaluated using the CIBERSORT bioinformatic algorithm. This algorithm, along with the R package “CIBERSORT” and the leukocyte gene signature matrix LM22, was employed to simulate and calculate the transcription feature matrix of 22 immune cells with 1,000 permutations and a significance threshold of p <0.05. Differences in the percentage of immune cells between IVDD and control samples were assessed using the Wilcoxon test, with P <0.05 to be statistically significant. The correlations between immune cells and potential biomarkers, as well as the correlations between different immune cells, were calculated using the R package “corrplot” and Pearson correlation analysis.

### 2.8 ROC analysis of the hub SRDEGs

The R package “pROC” was used for Receiver operating characteristics (ROC) analysis. Gene expression of the hub SRDEGs from dataset GSE23130 was analyzed using the “roc function”, and the “ci function” was used to evaluate the area under the curve (AUC) and confidence interval. In the ROC curve, sensitivity is on the Y-axis and false-positive rate (1−specificity) on the X-axis. An ROC curve with an AUC of at least 0.70 indicates sufficient predictive value.

### 2.9 RNA extraction and RT-qPCR

Eight nucleus pulposus tissue samples were collected at our hospital for PCR verification. According to the Pfirrmann grading system for disc degeneration, eight patients were divided into two groups: IVDD group (grades III–IV) and control group (grades I–II) (16). Written informed consent was obtained from all the patients. Total RNA was extracted from the nucleus pulposus tissue using the TRIzol reagent (Thermo Fisher Scientific). Prime-ScriptTM Master Mix (code no. RR036A) was used to reverse transcribe the total RNA into cDNA. Quantitative PCR was performed using SYBR^®^ Premix Ex Taq™ (Tli RNaseH Plus) on a LightCycler 480 II real-time fluorescence quantitative PCR instrument (ROCHE) with GAPDH as the internal control. Relative mRNA content was calculated using the 2^−ΔΔCt^ method. The primers used were as follows:

GAPDH forward primer (F): 5’-AGACACCATGGGGAAGGTGAA-3’, reverse primer (R): 5’-ATTGCTGATGATCTTGAGGCTG-3’;

MAPK1 forward primer (F): 5’-CTGCTGCTCAACACCACCT-3’, reverse primer (R): 5’-GCCACATATTCTGTCAGGAACC-3’;

FoxO1 forward primer (F): 5’-GAGGAGCCTCGATGTGGATG-3’, reverse primer (R): 5’-CCGAGATTTGGGGGAACGAA-3’;

ESR1 forward primer (F): 5’-TGGCTAGCGCTAGCTAGGGGGCTACA-3’, reverse primer (R): 5’-TGGGGCTAGGCTGCTCTAGCC-3’.

### 2.10 Statistical analysis

In the present study, statistical analyses were conducted using R 4.4.2 and GraphPad Prism 10.1.2. The unpaired Student’s t-test was applied to assess the difference between the IVDD and control groups, and a p-value of less than 0.05 was considered to be statistically significant.

## 3 Results

### 3.1 Determination of the senescence-related differentially expressed genes

**Figure 1:**
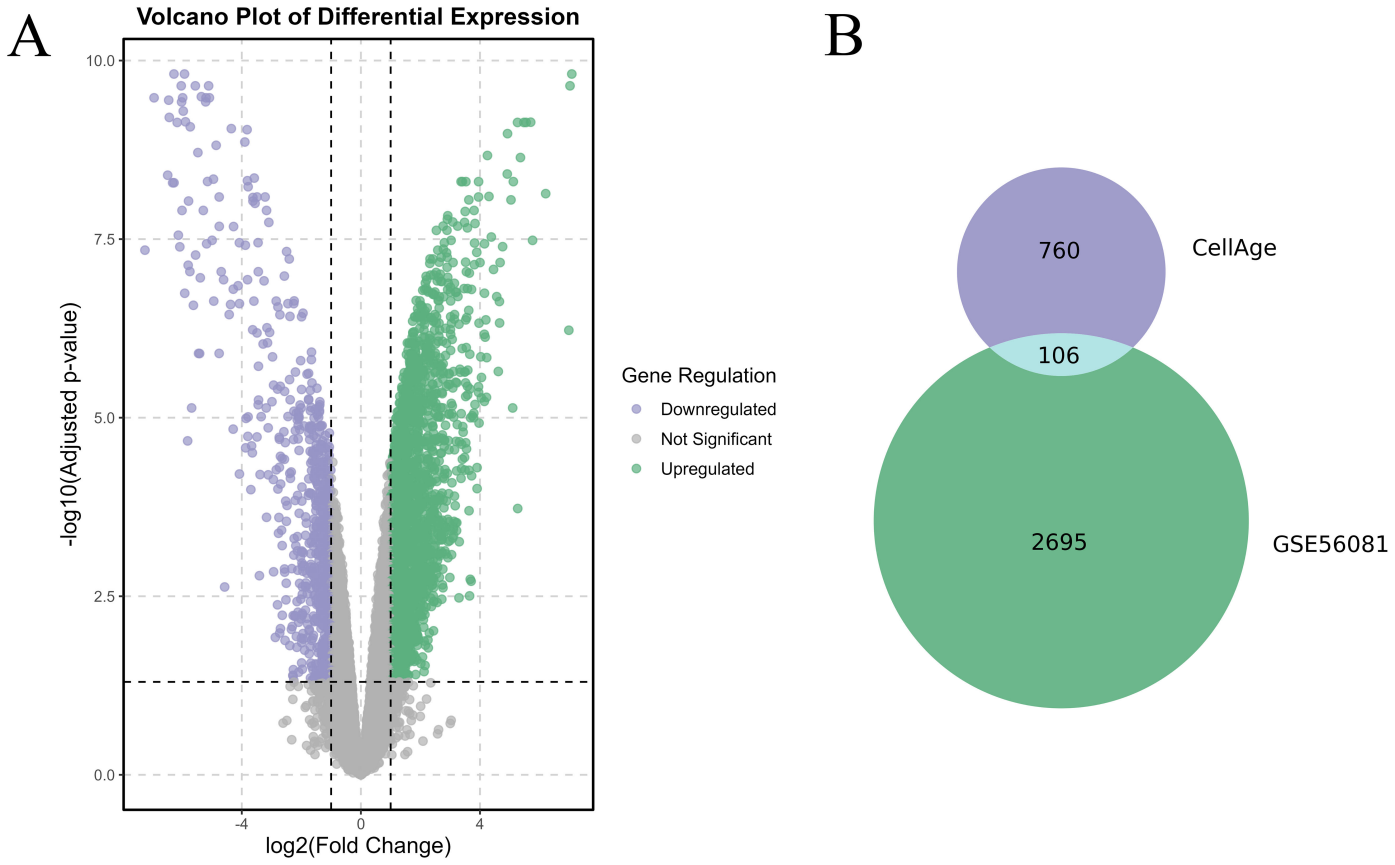
Identification of SRDEGs between IVDD and control groups. **(A)** Volcano plot of DEGs based on dataset GSE56081, where Y-axis is −log 10 (adjusted p value) and X-axis is log2 FC. Green dots represent upregulated genes and purple dots represent down-regulated genes. Gray dots represent genes that were not significantly differentially expressed. **(B)** Venn diagram based on DEGs and senescence-related genes based on CellAge database.

**Figure 2:**
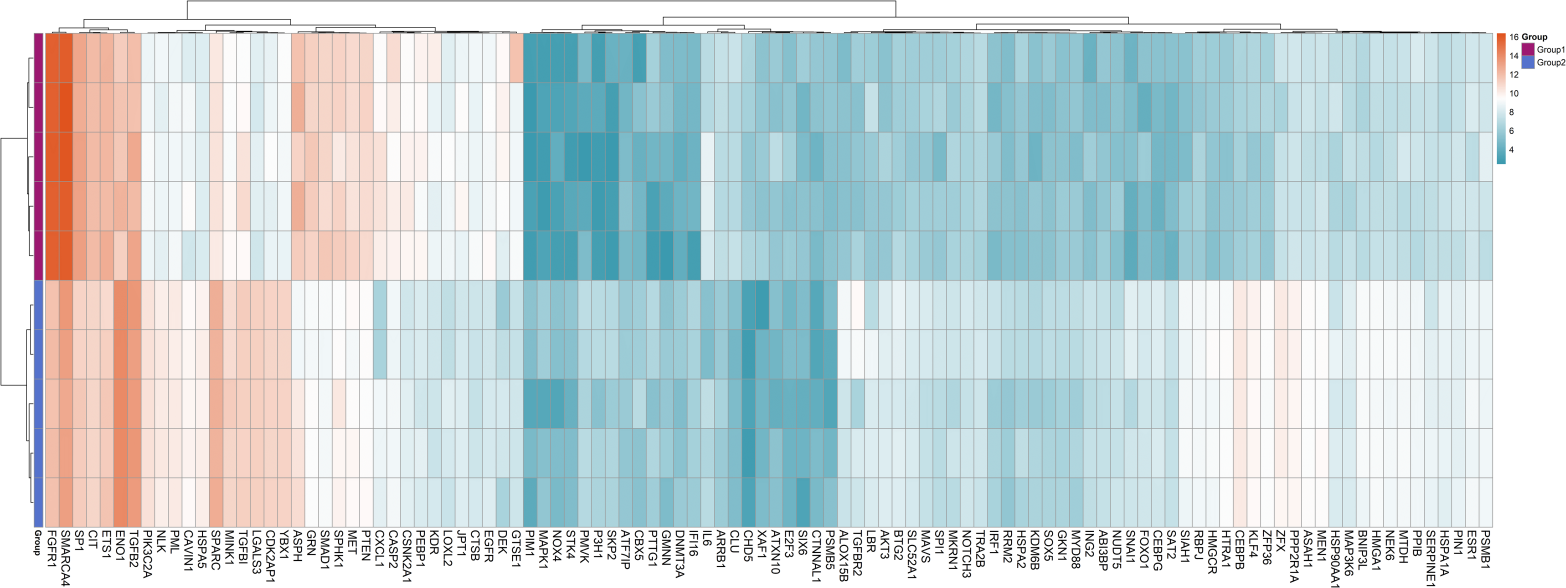
The heatmap of the 106 senescence-related differentially expressed genes (SRDEGs). Color scale represents the relative gene expression of each sample, the yellow represents high expression abundance and blue represents low expression abundance; Group 1 is the five samples from the control group and Group 2 is the five samples from the IVDD group.

Differentially expressed genes (DEGs) between the IVDD and control groups were identified using the GSE56081 dataset. A volcano plot (**Figure 1A**) showed 2,801 DEGs, with 2002 upregulated and 799 downregulated. We then intersected 866 senescence-related genes from the CellAge database with 2,801 DEGs and identified 106 senescence-related differentially expressed genes (SRDEGs) (**Figure 1B**), including 74 upregulated and 32 downregulated genes (**Table 1**). A hierarchical clustering heatmap demonstrated significant differences in SRDEG expression between the IVDD and control groups (**Figure 2**).

**Table 1:** List of the senescence-related differentially expressed genes.

Gene symbols	Expression	Number
FGFR1, SMARCA4, CHD5, GRN, CLU, SMAD1, SP1, PTEN, CIT, CSNK2A1, PEBP1, CTSB, EGFR, ARRB1, MET, ETS1, CASP2, DEK, SPHK1, ASPH, KDR, E2F3, JPT1, CXCL1, LOXL2, XAF1, GTSE1, PSMB5, IL6, CTNNAL1, ATXN10, SIX6	Downregulated	32
CEBPB, HTRA1, LGALS3, HMGCR, KLF4, PPP2R1A, RBPJ, ZFP36, ASAH1, SPARC, SIAH1, CEBPG, HSPA5, YBX1, BNIP3L, NEK6, CDK2AP1, SAT2, HMGA1, ZFX, SLC52A1, PIK3C2A, SKP2, ENO1, BTG2, AKT3, MEN1, NLK, PML, MTDH, P3H1, ABI3BP, SNAI1, FOXO1, NUDT5, HSPA1A, NOTCH3, ALOX15B, MINK1, PMVK, PPIB, ING2, STK4, MAVS, IFI16, TRA2B, PIN1, ESR1, MAP3K6, SOX5, MAPK1, KDM6B, SERPINE1, DNMT3A, GKN1, TGFBI, PIM1, LBR, TGFB2, PSMB1, ATF7IP, IRF1, TGFBR2, MYD88, MKRN1, NOX4, HSPA2, HSP90AA1, CBX5, SPI1, CAVIN1, RRM2, PTTG1, GMNN	Upregulated	74

### 3.2 GO and KEGG enrichment analysis

**Figure 3:**
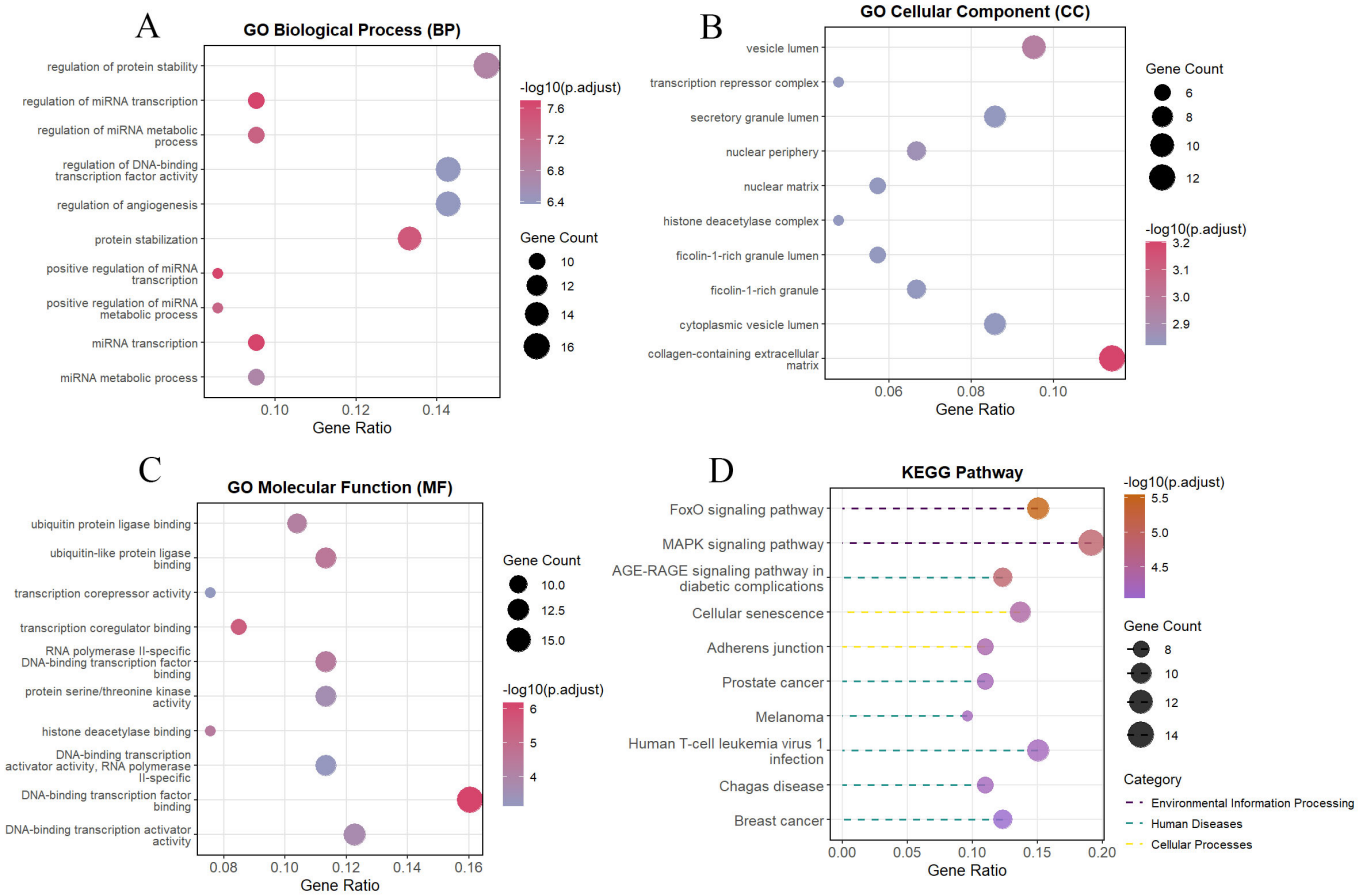
Functional enrichment analysis of SRDEGs. **(A)** The top 10 significantly enriched GO terms in the category of biological process (BP) for the SRDEGs; **(B)** The top 10 significantly enriched GO terms in the category of cellular component (CC); **(C)** The top 10 significantly enriched GO terms in the category of molecular function (MF); **(D)** Kyoto Encyclopedia of Genes and Genomes (KEGG) pathway enrichment analysis for the SRDEGs.

GO enrichment analysis indicated the following. In biological processes, SRDEGs were significantly enriched in terms of regulation of protein stability, regulation of miRNA transcription, and regulation of miRNA metabolism (**Figure 3A**). In the cellular components, SRDEGs were highly enriched in the vesicle lumen, transcription repressor complex, and secretory granule lumen (**Figure 3B**). In terms of the molecular function, SRDEGs were notably enriched in ubiquitin and ubiquitin-like ligase binding, transcriptional co-repressor activity, and transcriptional co-regulator binding (**Figure 3C**). KEGG enrichment analysis showed that SRDEGs were mainly associated with the FoxO, MAPK, and AGE-RAGE signaling pathways and cellular senescence (**Figure 3D**).

### 3.3 PPI network analysis and correlation analysis of the SRDEGs

**Figure 4:**
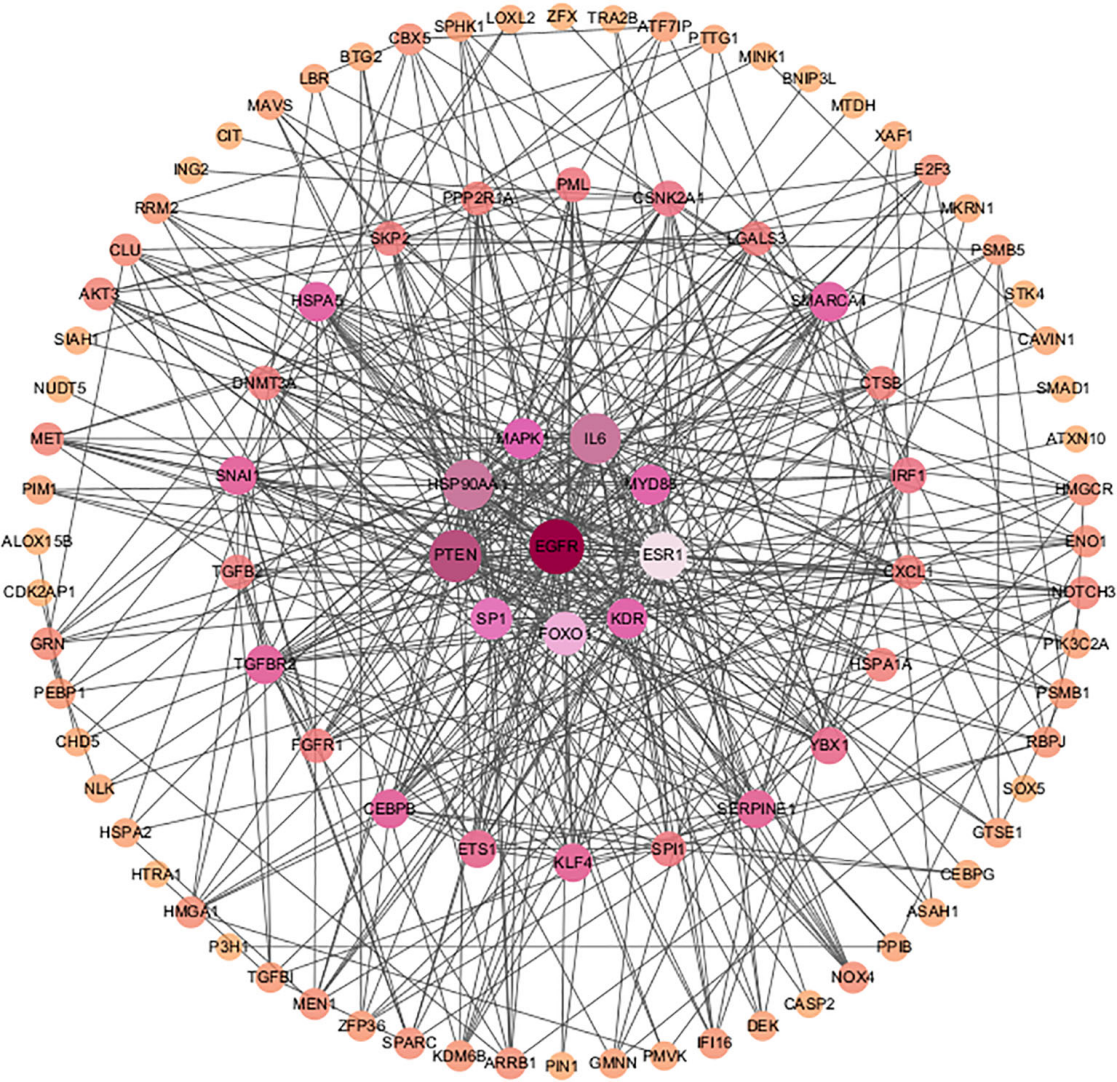
PPI network analysis of SRDEGs.

**Figure 5:**
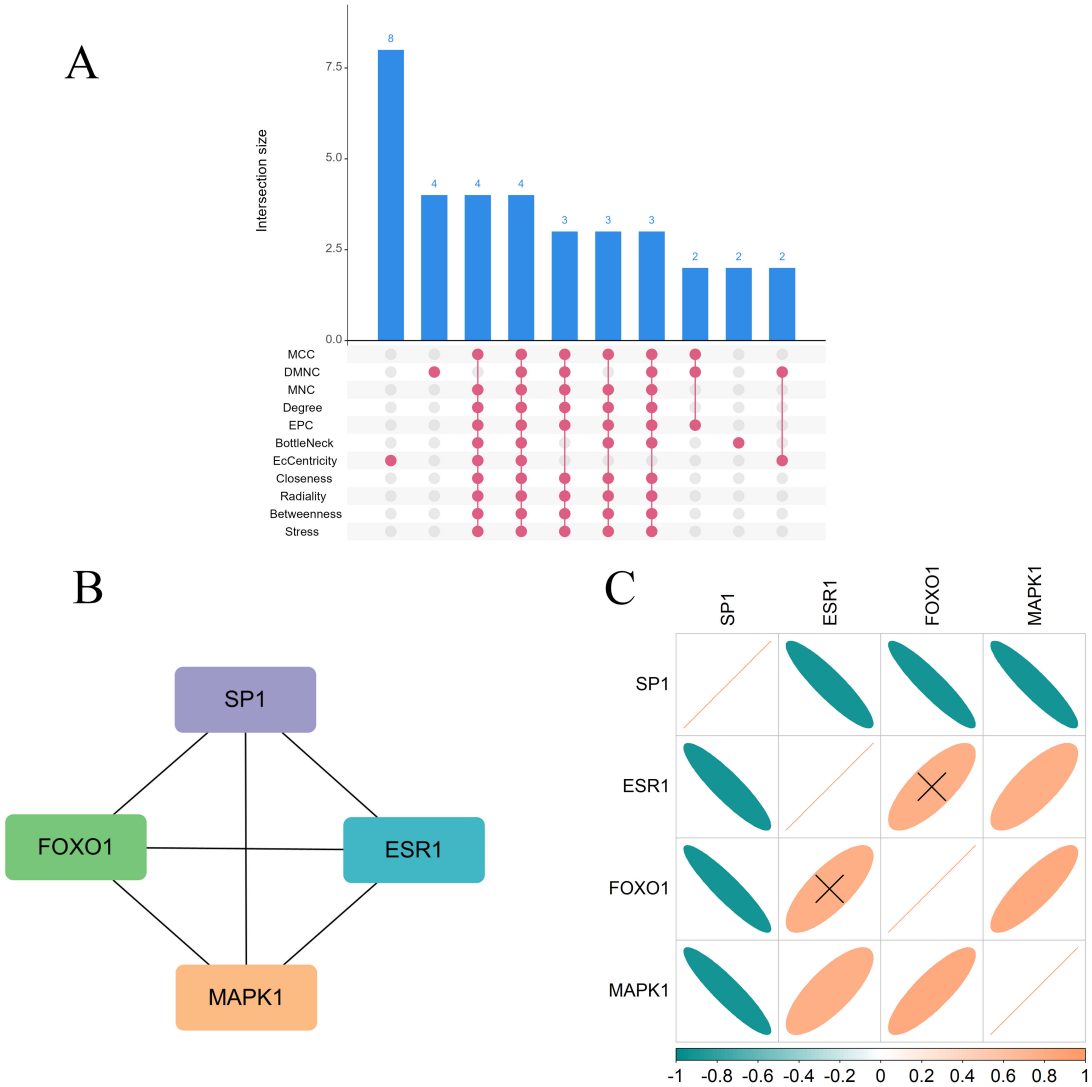
**(A)** Upset graph of eleven algorithms (CycloHubba MCC, DMNC, MNC, Degree, EPC, BottleNeck, EcCentricity, Closeness, Radiality, Betweenness, Stress); **(B)** Four hub SRDEGs: SP1, FoxO1, ESR1 and MAPK1; **(C)** A Pearson correlation analysis of four hub SRDEGs.

**Figure 6:**
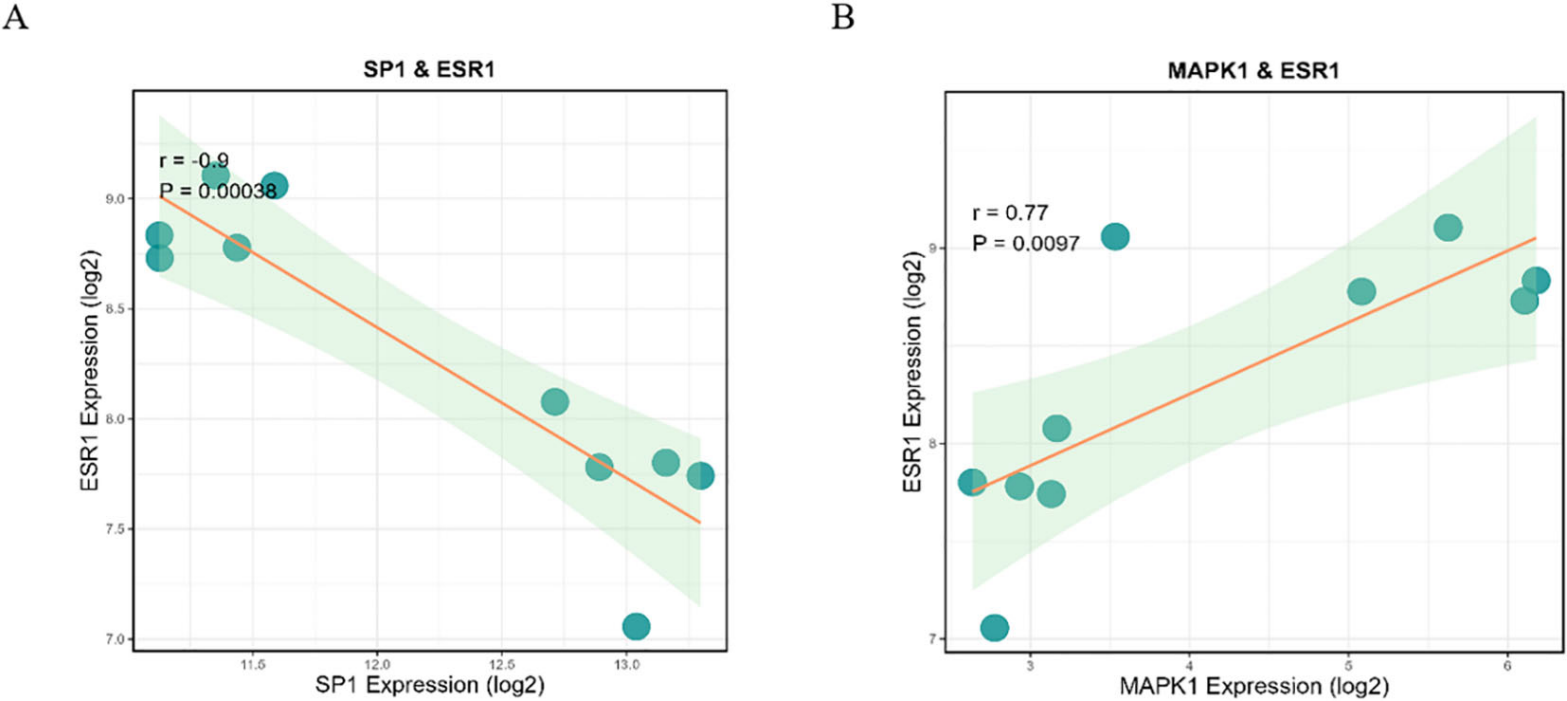
**(A)** Pearson correlation analysis showed that ESR1 expression was negatively correlated with SP1 expression. **(B)** Pearson correlation analysis showed that ESR1 expression was positively correlated with MAPK1 expression.

PPI network analysis based on the STRING database was performed and visualized using the Cytoscape software (**Figure 4**). The top 30 SRDEGs were then calculated using 11 algorithms (**Figure 5A**) in the CycloHubba plug-in, and the intersection was considered as the hub SRDEGs. Four hub SRDEGs were identified: SP1, FoxO1, ESR1, and MAPK1 (**Figure 5B**). SP1 was downregulated in IVDD, while the other three hub genes were upregulated (**Table 2**). In addition, Pearson correlation analysis was performed on the four hub SRDEGs (**Figure 5C**). It can be seen that the most negatively correlated is SP1-ESR1, and the most positively correlated is MAPK1-ESR1. Their correlation analysis is shown in **Figure 6A**, which shows a negative correlation between SP1 and ESR1 expression with an r value of −0.9 and p <0.01. **Figure 6B** shows that FoxO1 expression is positively correlated with MAPK1 expression with an r value of 0.77 and p <0.01.

**Table 2:** List of four hub SRDEGs.

Gene symbols	Expression	Log2 (fold change)	−log10 (adj.p.val)
SP1	Downregulated	−1.694	5.098
FOXO1	Upregulated	2.798	4.593
ESR1	Upregulated	1.210	3.338
MAPK1	Upregulated	2.376	2.876

### 3.4 Function analysis of the hub SRDEGs

**Figure 7:**
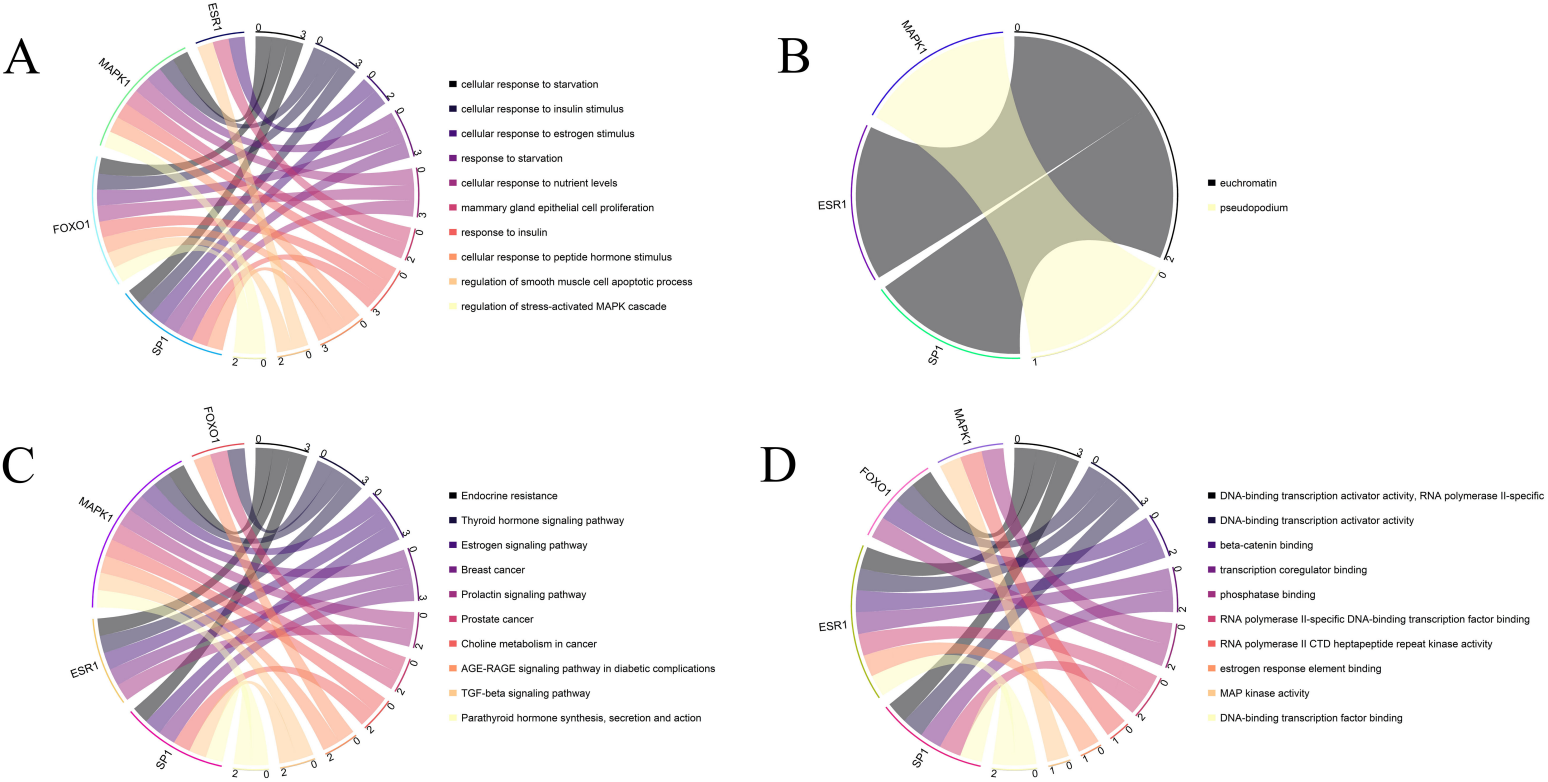
Functional enrichment analysis of hub SRDEGs. **(A)** The top 10 significantly enriched GO terms in the category of biological process for these hub SRDEGs; **(B)** The top 10 significantly enriched GO terms in the category of cellular component; **(C)** The top 10 significantly enriched GO terms in the category of molecular function; **(D)** KEGG pathway analysis for the hub SRDEGs.

GO enrichment analysis revealed that the hub SRDEGs were mainly enriched in cellular response to starvation, cellular response to insulin stimulus, cellular response to estrogen stimulus, and cellular response to nutrient levels in biological processes (**Figure 7A**), chromatin and pseudopodia in cellular components (**Figure 7B**), and DNA-binding transcriptional activator activity, transcriptional coregulator binding, and MAP kinase activity in molecular functions (**Figure 7C**). KEGG enrichment analysis indicated that the key SRDEGs were mainly associated with the AGE-RAGE, TNF-β, and prolactin signaling pathways (**Figure 7D**).

### 3.5 Immune infiltration analysis

**Figure 8:**
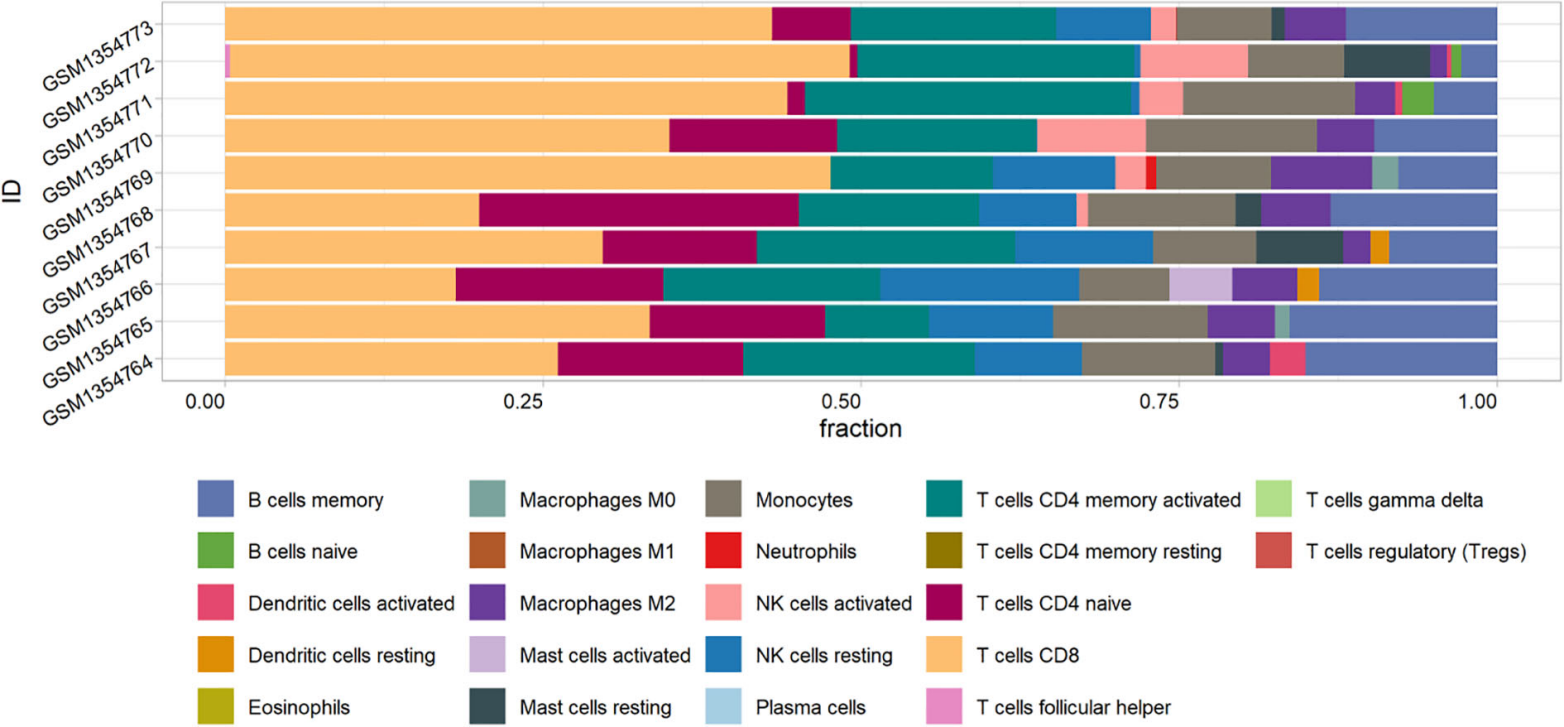
The proportion of 22 immune cells in samples in GSE56081. The x-axis represents the proportion of 22 immune cells, and the y-axis represents the 10 samples from GSE56081.

**Figure 9:**
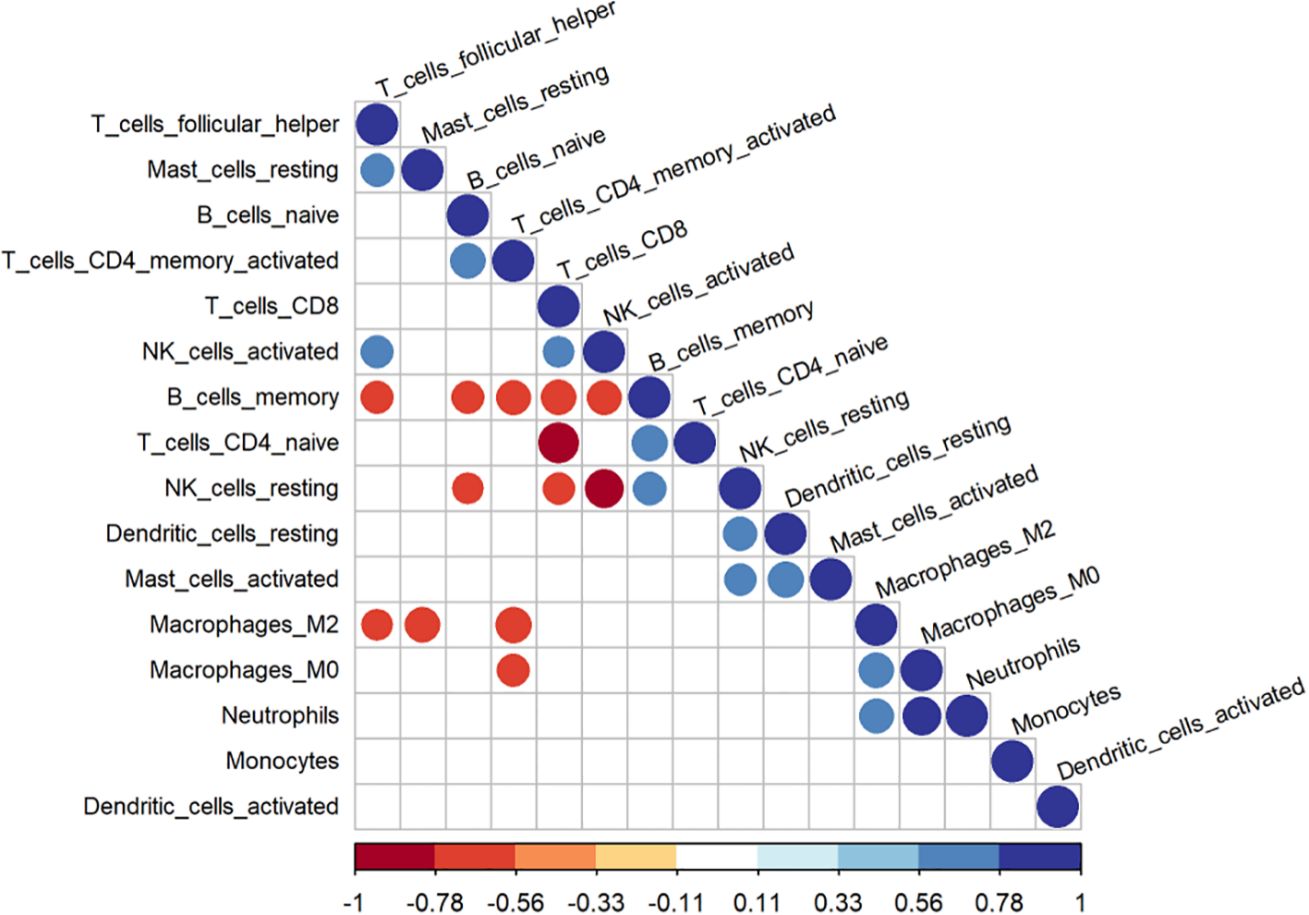
The correlations of 22 immune cells. The redder the color, the stronger the negative correlation; the bluer the color, the stronger the positive correlation.

**Figure 10:**
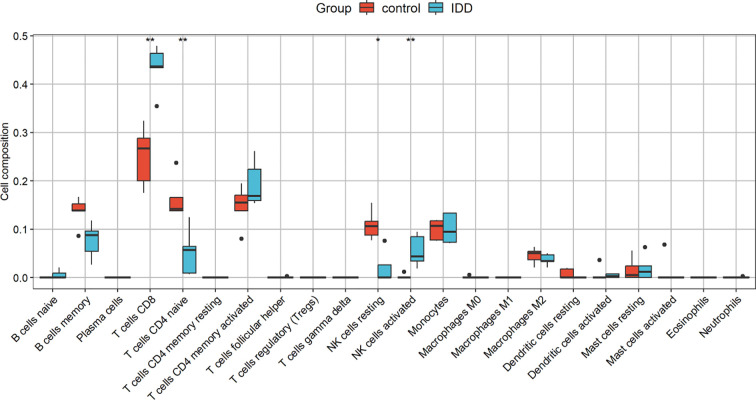
Boxplot of the difference in the proportion of immune cells between the IVDD group and the control group. The x-axis represents the 22 types of immune cells, and the y-axis represents the proportion of cellular composition in the samples. The levels of naive CD4 T cells and resting NK cells were relatively high in the control group, and the levels of CD8 T cells and activated NK cells were higher in the IVDD group. *p <0.05, **p <0.01.

**Figure 8** shows the proportion of 22 immune cells in the samples using a bar chart. As indicated by the CIBERSORT algorithm, neutrophils and M0 macrophages showed the most significant positive correlation. In contrast, CD8 T cells and naive CD4 T cells displayed the strongest negative correlation (**Figure 9**). A box plot of immune cells (**Figure 10**) revealed that the control group had relatively higher levels of naive CD4 T cells and resting NK cells. Conversely, the IVDD groups exhibited higher quantities of CD8 T cells and activated NK cells, which may be related to the IVDD process.

**Figure 11:**
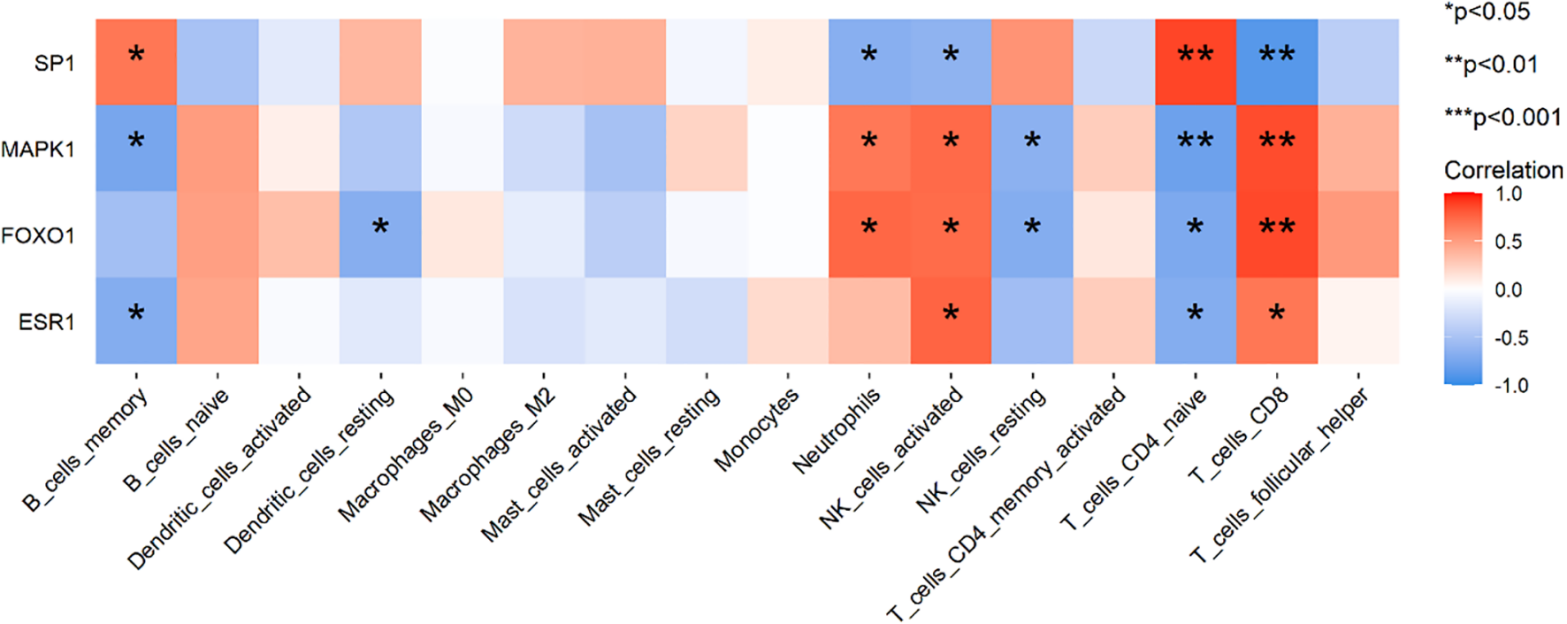
The correlations between the hub SRDEGs and immune cells. The x-axis represents the 22 types of immune cells, and the y-axis represents the four hub SRDEGs. Red indicates positive correlation (darker red signifies stronger positive correlation), while blue indicates negative correlation (darker blue signifies stronger negative correlation). *p <0.05, **p <0.01.

In addition, the correlations between the hub SRDEGs and immune cells were as follows: SP1 was positively correlated with naive CD4 T cells and B memory cells and negatively correlated with CD8 T cells, activated NK cells, and neutrophils. MAPK1 is negatively correlated with naive CD4 T cells, B memory cells, and resting NK cells, whereas it is positively correlated with CD8 T cells, activated NK cells, and neutrophils. FoxO1 is negatively associated with naive CD4 T cells, resting dendritic cells, and resting NK cells and positively associated with CD8 T cells, activated NK cells, and neutrophils. ESR1 was negatively correlated with naive CD4 T cells and B memory cells and positively correlated with CD8 T cells and activated NK cells (**Figure 11**).

### 3.6 The validation of hub SRDEGs in GSE23130 dataset

**Figure 12:**
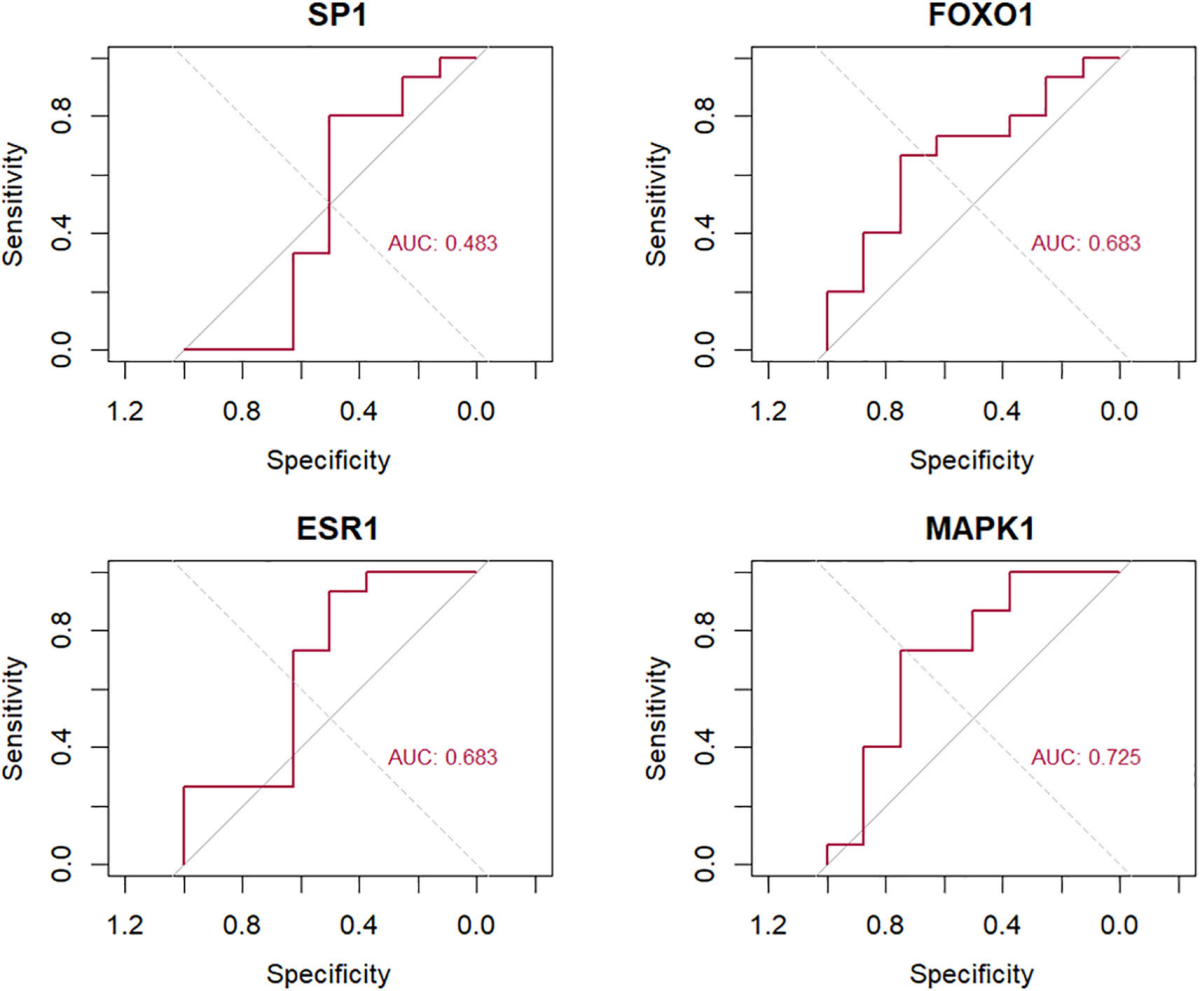
ROC curve of hub SRDEGs based on GSE23130 dataset, including SP1, FOXO1, ESR1, and MAPK1. The X-axis represents the (1 − Specificity), and the Y-axis represents the Sensitivity.

Based on the dataset GSE23130, the hub SRDEGs were verified using the ROC curve, as shown in **Figure 12**. The AUC for SP1 is 0.483, FoxO1 is 0.683, ESR1 is 0.683, and MAPK1 is 0.725, which is above the threshold of 0.7.

### 3.7 Expression validation of hub SRDEGs

**Figure 13:**
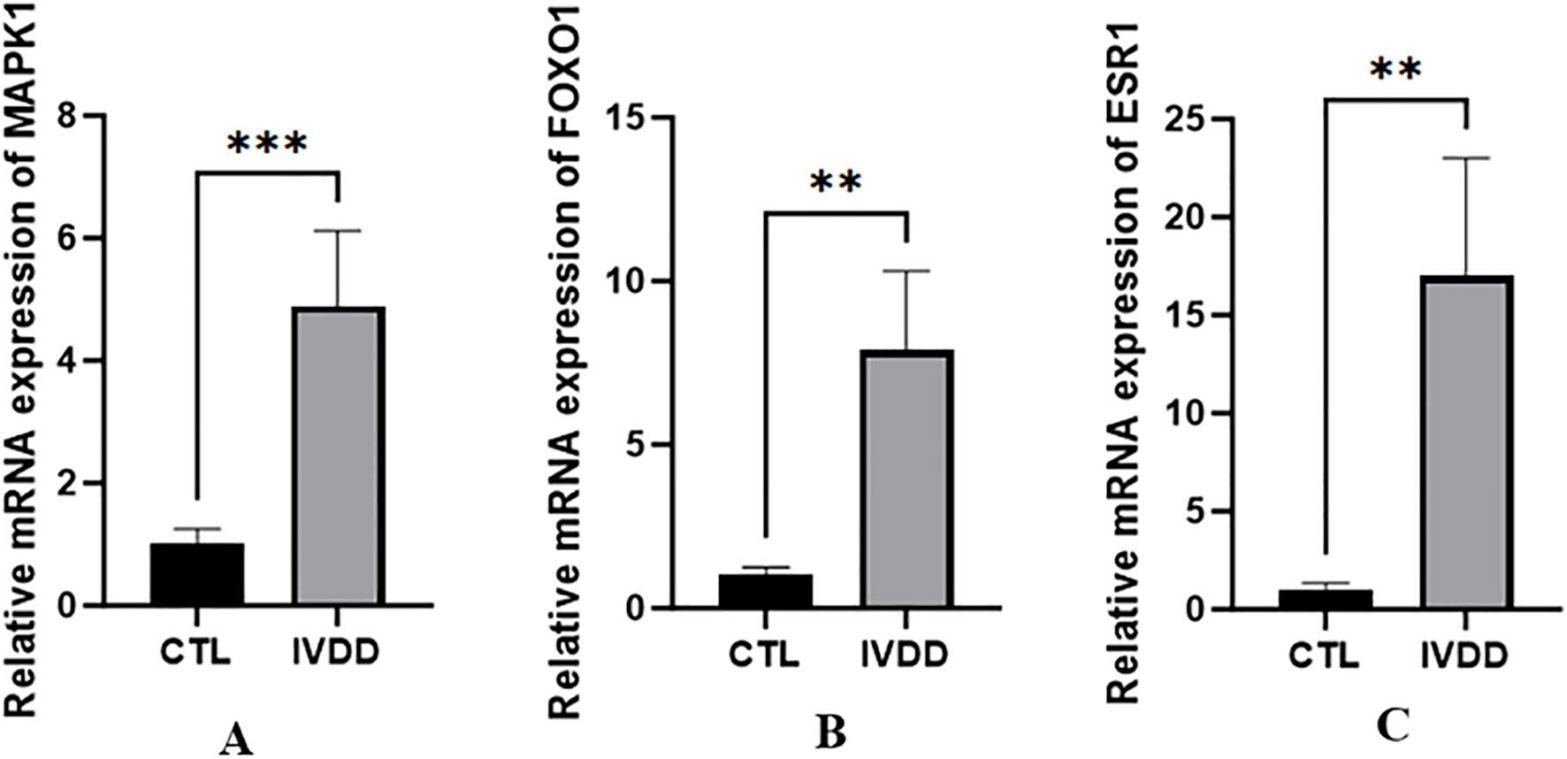
The expression differences of MAPK1, FoxO1 and ESR1 genes between IVDD group and control group. **p <0.01, ***p <0.001.

RT-qPCR confirmed the difference in MAPK1 expression in the nucleus pulposus between the degeneration and control groups, showing higher MAPK1 expression in the IVDD group (t = 3.229, P <0.001). FoxO1 and ESR1 expression was also confirmed to be higher in the IVDD group (t = 5.675, P = 0.0013; t = 5.341, P = 0.0018, respectively) (**Figure 13**).

## 4 Discussion

Intervertebral disc degeneration (IVDD), marked by nucleus pulposus cell reduction and extracellular matrix degradation, is a major cause of lower back pain and many degenerative diseases, posing a heavy global healthcare burden (17, 18). However, the pathogenesis of this condition remains controversial. Oxidative stress, inflammatory conditions, abnormal mechanical stress, and nutritional metabolic imbalance are contributing factors. Cell senescence plays a key role in these mechanisms and is increasingly being found to be closely related to the occurrence and development of degenerative disc diseases (9, 19, 20).

The present study analyzed GSE56081 datasets using bioinformatics and found 2,801 DEGs, with 2,002 upregulated and 799 downregulated genes. Intersection with senescence-related genes yielded 106 SRDEGs (74 upregulated and 32 downregulated). GO function analysis showed that SRDEGs were mainly involved in regulating protein stability, miRNA transcription, and miRNA metabolism in biological process. Intracellular protein degradation is primarily maintained through the ubiquitin-proteasome system (UPS) and autophagy. In senescent cells, the decline in UPS function and autophagy efficiency leads to the accumulation of misfolded or oxidatively damaged proteins, activating the unfolded protein response and oxidative stress, and ultimately triggering cell cycle arrest (21, 22). Additionally, the significant enrichment of ubiquitin and ubiquitin-like ligase binding in molecular functions further supports the above viewpoints. KEGG pathway analysis showed that SRDEGs were significantly enriched in the FoxO, MAPK, and AGE-RAGE signaling pathways, which are crucial for cellular senescence.

The FoxO transcription factor family consists of four members, among which FoxO1/3/4 are all involved in senescence-related signaling pathways that influence cellular development, metabolism, and lifespan.

The FoxO signaling pathway is primarily regulated by protein kinase B (PKB/Akt) and mitogen-activated protein kinases (MAPK). Nogueira et al. (23) demonstrated that in mouse embryonic fibroblasts, Akt acts as a senescence-inducing agent by inactivating FoxO1 and FoxO3, including in contexts of replicative senescence, oxidative stress-induced senescence, and oncogenic Ras-induced senescence. The MAPK signaling pathway comprises four major branches, ERK, JNK, p38/MAPK, and ERK5 (24), all of which play critical roles in cellular senescence. The MAPK/ERK pathway (25), a classical pro-survival signaling cascade, is closely linked to the differentiation of mesenchymal stem cells (MSCs). Inhibition of ERK blocks spontaneous MSC differentiation, whereas ERK1/2 suppresses apoptosis. Furthermore, the MAPK/ERK signaling pathway regulates the expression of downstream target genes, such as p16 and p53, with p16 being a key senescence-associated gene (26). Xie et al. (27) demonstrated that ERK, JNK, and p38/MAPK collectively exert significant effects during induced senescence in testicular stem Leydig cells. The AGE-RAGE signaling pathway refers to a cascade triggered by the binding of advanced glycation end products (AGEs), glycated proteins resulting from hyperglycemia, to their receptor RAGE, ultimately activating downstream cellular responses (28). Ferrucci et al. (29) identified the most prominent aging-associated metabolic pathways, including IGF signaling, MAPK signaling, HIF-1 signaling, cytokine signaling, FoxO metabolic pathways, folate metabolism, and AGE-RAGE-related metabolic pathways.

To further identify the hub genes associated with cellular senescence in intervertebral disc degeneration, PPI network analysis combined with 11 computational algorithms identified four hub genes: SP1, FoxO1, ESR1, and MAPK1. Among these, SP1 was downregulated in IVDD, whereas the other three genes (FoxO1, ESR1, and MAPK1) exhibited upregulated expression. External dataset validation using ROC curves revealed the following AUC values: SP1, 0.483; FoxO1, 0.683; ESR1, 0.683; and MAPK1, 0.725. Interestingly, while SP1 was identified as a hub gene based on its high centrality in the SRDEG PPI network, suggesting a key structural role in potential senescence-related interactions, its diagnostic value, assessed by ROC analysis on the validation dataset, was low (AUC = 0.483). This discrepancy highlights the fact that topological importance within a network (hub status) does not always translate directly into strong discriminatory power based on expression levels alone (diagnostic value). The functional impact of SP1 may depend more on changes in activity or post-translational modifications rather than significant alterations in mRNA abundance. RT-qPCR confirmed that MAPK1 expression was higher in the IVDD group. These results showed that MAPK1 may be a key driver of cellular senescence, contributing to IVDD progression. The intervertebral disc is an immune-privileged tissue in the body, covered by the annulus fibrosus and upper and lower cartilaginous endplates (3, 30). However, when nucleus pulposus cells (NPCs) degenerate, they disrupt the NPC-blood barrier, directly exposing NPCs to the host’s immune system. This ultimately triggers immune responses and massive infiltration of immune cells, including macrophages, neutrophils, mast cells, CD4 T cells, CD8 T cells, Treg cells, and B cells (7). In the present study, immune cell infiltration analysis showed that the expression levels of naive CD4 T cells and resting NK cells were higher in the control group, while CD8 T cells and activated NK cells were higher in the IVDD groups, which play pivotal roles in immune-related inflammatory responses associated with IVDD. The correlations between MAPK1 and immune cells showed that MAPK1 was negatively correlated with naive CD4 T cells, B memory cells, and resting NK cells, whereas it was positively correlated with CD8 T cells, activated NK cells, and neutrophils.

The MAPK1 gene, also termed ERK or ERK2, is located on human chromosome 22q11.22, and encodes the ERK2 protein, a core component of the MAPK/ERK signaling pathway. As mentioned previously, the MAPK/ERK pathway (25) is a classical pro-survival signaling cascade that is closely related to the differentiation of MSCs. ERK2 plays an essential role in fundamental biological processes, including cell proliferation, differentiation, survival, senescence, and stress responses. Nojima et al. (31) found that in mouse embryonic fibroblasts, downregulation of IGFBP5 promoted replicative senescence by activating ERK2. Furthermore, in IGFBP5-knockdown cells, silencing of ERK2, but not ERK1, prevented an increase in SA-β-gal-positive cells. Shin et al. (32) also found that the depletion of ERK2, but not ERK1, abrogates oncogenic Ras-induced senescence. Furthermore, ERK2 regulates the expression of the downstream target gene p16, a key gene involved in cell senescence (26, 31). MAPK1/ERK2 exhibits dual regulatory effects in cellular senescence: in normal cells, ERK2 delays senescence by promoting proliferation and inhibiting apoptosis, whereas in senescent cells, its sustained activation may negatively regulate the MAPK pathway through feedback mechanisms and crosstalk with other pathways, such as PI3K/AKT and p53 signaling, ultimately inducing cell cycle arrest (33–35). Furthermore, ERK2 modulates cellular senescence by regulating telomerase activity and telomere length (36). The MAPK cascade plays diverse roles in immune responses, including initiation of innate immunity, activation of adaptive immunity, and regulation of immunogenic cell death (37). ERK2 is a critical upstream regulator of neuroinflammatory pathologies. In demyelinating mouse models, ERK2 activation exacerbates inflammatory demyelination by inducing proinflammatory mediators and gliosis, whereas ERK2-deficient mice exhibit reduced levels of these inflammatory mediators (38). The role of ERK2 in immune responses during intervertebral disc degeneration remains poorly understood, and future studies should focus on elucidating its regulatory mechanisms in immune cell infiltration, pro-inflammatory cytokine production, and extracellular matrix degradation within the disc microenvironment.

The present study had some limitations. First, this study predominantly relied on bioinformatic analyses, with only preliminary RT-qPCR validation performed for hub genes. Protein-level validation, such as western blotting, was not performed to assess total and phosphorylated MAPK1/ERK2. Additionally, the lack of functional assays, such as siRNA knockdown of MAPK1 followed by senescence marker quantification, SASP-related gene expression profiling, or co-culture assays with immune cells, makes it difficult to draw definitive conclusions regarding the causal role of MAPK1 in cellular senescence and immune infiltration. Future studies that incorporate these missing experimental validations are essential to confirm the proposed mechanism.

## 5 Conclusion

In conclusion, the present study employed bioinformatics techniques to investigate the mechanisms of cellular senescence and immune infiltration in intervertebral disc degeneration. By integrating multiple algorithms to screen for senescence-related genes in IVDD, our findings highlight MAPK1 as a critical factor that plays a pivotal role in regulating both senescence processes in nucleus pulposus cells and immune infiltration mechanisms.

## Data Availability

Publicly available datasets were analyzed in this study. This data can be found here: GSE56081 and GSE23130.
