# Supplementary material for: Identification and validation of cellular senescence-related genes and immune cell infiltration characteristics in intervertebral disc degeneration
Source: Front Immunol. 2025 May 29;16:1589849. doi: 10.3389/fimmu.2025.1589849 (PMC12158716; doi:10.3389/fimmu.2025.1589849)
Supplement: Supplementary file 1 [file DataSheet1.pdf]

Downregulated Genes - 32:

1. **FGFR1** - fibroblast growth factor receptor 1
2. **SMARCA4** - SWI/SNF related, matrix associated, actin dependent regulator of chromatin, subfamily a, member 4
3. **CHD5** - chromodomain helicase DNA binding protein 5
4. **GRN** - granulin precursor
5. **CLU** - clusterin
6. **SMAD1** - SMAD family member 1
7. **SP1** - Sp1 transcription factor
8. **PTEN** - phosphatase and tensin homolog
9. **CIT** - citron rho-interacting serine/threonine kinase
10. **CSNK2A1** - casein kinase 2 alpha 1
11. **PEBP1** - phosphatidylethanolamine binding protein 1
12. **CTSB** - cathepsin B
13. **EGFR** - epidermal growth factor receptor
14. **ARRB1** - arrestin beta 1
15. **MET** - MET proto-oncogene, receptor tyrosine kinase
16. **ETS1** - ETS proto-oncogene 1, transcription factor
17. **CASP2** - caspase 2
18. **DEK** - DEK proto-oncogene
19. **SPHK1** - sphingosine kinase 1
20. **ASPH** - aspartate beta-hydroxylase
21. **KDR** - kinase insert domain receptor
22. **E2F3** - E2F transcription factor 3
23. **JPT1** - Jupiter microtubule associated homolog 1
24. **CXCL1** - C-X-C motif chemokine ligand 1
25. **LOXL2** - lysyl oxidase like 2
26. **XAF1** - XIAP associated factor 1
27. **GTSE1** - G2 and S phase expressed 1
28. **PSMB5** - proteasome 20S subunit beta 5
29. **IL6** - interleukin 6
30. **CTNNAL1** - catenin alpha like 1
31. **ATXN10** - ataxin 10
32. **SIX6** - SIX homeobox 6

Upregulated Genes - 74:

1. **CEBPB** - CCAAT enhancer binding protein beta
2. **HTRA1** - HtrA serine peptidase 1
3. **LGALS3** - galectin 3
4. **HMGCR** - 3-hydroxy-3-methylglutaryl-CoA reductase
5. **KLF4** - KLF transcription factor 4
6. **PPP2R1A** - protein phosphatase 2 scaffold subunit Aalpha
7. **RBPJ** - recombination signal binding protein for immunoglobulin kappa J region
8. **ZFP36** - ZFP36 ring finger protein
9. **ASAH1** - N-acylsphingosine amidohydrolase 1

10. **SPARC** - secreted protein acidic and rich in cysteine
11. **SIAH1** - siah E3 ubiquitin protein ligase 1
12. **CEBPG** - CCAAT enhancer binding protein gamma
13. **HSPA5** - heat shock protein family A (Hsp70) member 5
14. **YBX1** - Y-box binding protein 1
15. **BNIP3L** - BCL2 interacting protein 3 like
16. **NEK6** - NIMA related kinase 6
17. **CDK2AP1** - cyclin dependent kinase 2 associated protein 1
18. **SAT2** - spermine/spermidine N1-acetyltransferase family member 2
19. **HMGA1** - high mobility group AT-hook 1
20. **ZFX** - ZFY related X-linked zinc finger protein
21. **SLC52A1** - solute carrier family 52 member 1
22. **PIK3C2A** - phosphatidylinositol-4-phosphate 3-kinase catalytic subunit type 2 alpha
23. **SKP2** - S-phase kinase associated protein 2
24. **ENO1** - enolase 1
25. **BTG2** - BTG anti-proliferation factor 2
26. **AKT3** - AKT serine/threonine kinase 3
27. **MEN1** - menin 1
28. **NLK** - nemo like kinase
29. **PML** - PML nuclear body scaffold
30. **MTDH** - metadherin
31. **P3H1** - prolyl 3-hydroxylase 1
32. **ABI3BP** - ABI family member 3 binding protein
33. **SNAI1** - snail family transcriptional repressor 1
34. **FOXO1** - forkhead box O1
35. **NUDT5** - nudix hydrolase 5
36. **HSPA1A** - heat shock protein family A (Hsp70) member 1A
37. **NOTCH3** - notch receptor 3
38. **ALOX15B** - arachidonate 15-lipoxygenase type B
39. **MINK1** - misshapen like kinase 1
40. **PMVK** - phosphomevalonate kinase
41. **PPIB** - peptidylprolyl isomerase B
42. **ING2** - inhibitor of growth family member 2
43. **STK4** - serine/threonine kinase 4
44. **MAVS** - mitochondrial antiviral signaling protein
45. **IFI16** - interferon gamma inducible protein 16
46. **TRA2B** - transformer 2 beta homolog
47. **PIN1** - peptidylprolyl cis/trans isomerase, NIMA-interacting 1
48. **ESR1** - estrogen receptor 1
49. **MAP3K6** - mitogen-activated protein kinase kinase kinase 6
50. **SOX5** - SRY-box transcription factor 5
51. **MAPK1** - mitogen-activated protein kinase 1
52. **KDM6B** - lysine demethylase 6B
53. **SERPINE1** - serpin family E member 1

54. **DNMT3A** - DNA methyltransferase 3 alpha
55. **GKN1** - gastrokine 1
56. **TGFBI** - transforming growth factor beta induced
57. **PIM1** - Pim-1 proto-oncogene, serine/threonine kinase
58. **LBR** - lamin B receptor
59. **TGFB2** - transforming growth factor beta 2
60. **PSMB1** - proteasome 20S subunit beta 1
61. **ATF7IP** - activating transcription factor 7 interacting protein
62. **IRF1** - interferon regulatory factor 1
63. **TGFBR2** - transforming growth factor beta receptor 2
64. **MYD88** - MYD88 innate immune signal transduction adaptor
65. **MKRN1** - makorin ring finger protein 1
66. **NOX4** - NADPH oxidase 4
67. **HSPA2** - heat shock protein family A (Hsp70) member 2
68. **HSP90AA1** - heat shock protein 90 alpha family class A member 1
69. **CBX5** - chromobox 5
70. **SPI1** - Spi-1 proto-oncogene
71. **CAVIN1** - caveolae associated protein 1
72. **RRM2** - ribonucleotide reductase regulatory subunit M2
73. **PTTG1** - PTTG1 regulator of sister chromatid separation, securin
74. **GMNN** - geminin DNA replication inhibitor
